# 

**Published:** 1875-01

**Authors:** 


### Books and Pamphlets Received.

A Sketch of the Early History of Practical Anatomy. The Introductory Address to the Course of Lectures on Anatomy at the Philadelphia School of Anatomy. By W. W. Keen, M. D. Philadelphia: J. B. Lippincott & Co., 1874. Buffalo: T. Butler & Son.

Experimentation on Animals as a means of knowledge of Physiology, Pathology and Practical Medicine. By J. C. Dalton, M. D. New York: F. W. Christern.

Contributions to the Annals of Medical Progress and Medical Education in the United States, before the war of Independence. By Joseph M. Toner, M. D.

Chimes for Childhood, or a Collection of Songs for little ones. Boston: Estes & Lauriat. Buffalo: Martin Taylor.

Eulogy on Chief Justice Chase, delivered by William M. Evarts, before the Alumni of Dartmouth College. Boston: Estes & Lauriat, 1874. Buffalo Martin Taylor.

On Reflex Irritation of the Genito-Urinary Tract, resulting from Contraction of the Urethra at or near the Meatus Urinarius, either congenital or acquired. By F. N. Otis, M. D.

Transactions of the Medical Society of the District of Columbia, January, 1875.

Report of the New York City Council of Political Reform, for the years 1872–3 and 74, with Summary.

On Deaf-Mutism and the Method of Educating the Deaf and Dumb. By Laurence Tumbull, M. D. From the Transactions of the Pennsylvania State Medical Society.

THE BUFFALO

### Medical and Surgical Journal

PUBLISHED MONTHLY,

Containing Original Articles, Reports of Medical Societies and Hospitals, Editorial, Reviews, Correspondence, Army News, etc., etc; including the usual variety of Medical Periodical Publications. Specimen copies sent on application.

Address Buffalo Medical & Surgical Journal, Buffalo, N. Y.

TERMS OF SUBSCRIPTION, - - $3.00 PER YEAR, IN ADVANCE.

